# Clinical outcomes of implantable cardioverter-defibrillator therapy in noncompaction cardiomyopathy: a systematic review and meta-analysis

**DOI:** 10.1007/s10741-022-10250-w

**Published:** 2022-06-10

**Authors:** Martijn Tukker, Arend F. L. Schinkel, Adem Dereci, Kadir Caliskan

**Affiliations:** Thoraxcenter, Department of Cardiology, Erasmus MC University Medical Center Rotterdam, Room RG 431, Dr. Molewaterplein 40, 3015 GD Rotterdam, the Netherlands

**Keywords:** Noncompaction cardiomyopathy, Implantable cardioverter-defibrillator (ICD), Sudden cardiac death, Systematic review and meta-analysis, Appropriate ICD treatment, Mortality

## Abstract

A subgroup of patients with noncompaction cardiomyopathy (NCCM) is at increased risk of ventricular arrhythmias and sudden cardiac death (SCD). In selected patients with NCCM, implantable cardioverter-defibrillator (ICD) therapy could be advantageous for preventing SCD. Currently, there is no complete overview of outcome and complications after ICD therapy in patients with NCCM. This study sought to present an overview using pooled data of currently available studies. Embase, MEDLINE, Web of Science, and Cochrane databases were searched and returned 915 studies. After a thorough examination, 12 studies on outcome and complications after ICD therapy in patients with NCCM were included. There were 275 patients (mean age 38.6 years; 47% women) with NCCM and ICD implantation. Most of the patients received an ICD for primary prevention (66%). Pooled analysis demonstrates that the appropriate ICD intervention rate was 11.95 per 100 person-years and the inappropriate ICD intervention rate was 4.8 per 100 person-years. The cardiac mortality rate was 2.37 per 100 person-years. ICD-related complications occurred in 10% of the patients, including lead malfunction and revision (4%), lead displacement (3%), infection (2%), and pneumothorax (2%). Patients with NCCM who are at increased risk of SCD may significantly benefit from ICD therapy, with a high appropriate ICD therapy rate of 11.95 per 100 person-years and a low cardiac mortality rate of 2.37 per 100 person-years. Inappropriate therapy rate of 4.8 per 100 person-years and ICD-related complications were not infrequent and may lead to patient morbidity.

**Supplementary information:**

The online version contains supplementary material available at 10.1007/s10741-022-10250-w.

## Introduction

Noncompaction cardiomyopathy (NCCM) is characterized by hypertrabeculation of the ventricular myocardial wall and is associated with an increased risk of sudden cardiac death (SCD) [1, 2].

The etiology of NCCM is multivariable; a genetic substrate is reported in approximately 48% of the patients [3, 4].

Initial studies reported ventricular arrhythmias in up to 47% of the adult NCCM patients and sudden cardiac death in 18% [1, 2]. Current treatment of patients with NCCM consists of managing heart failure, supraventricular and ventricular arrhythmias, and preventing SCD [5]. In selected patients with NCCM, that are increased risk of ventricular arrhythmias, an implantable cardioverter-defibrillator (ICD) is a rational option. Currently, the information on ICD treatment specifically in patients with NCCM is limited. The aim of this systematic review and meta-analysis is to provide an overview of clinical outcome, complications, and appropriate and inappropriate ICD therapy in patients with NCCM. The information is clinically relevant and can be used during counseling and risk stratification of patients with NCCM.

## Methods

### Protocol and guidelines

A review protocol was written prior to the conduction of this research, and it can be found in the Online Resource. The Preferred Reporting Items for Systematic Reviews and Meta-Analyses guidelines were utilized for this systematic review [6].

### Eligibility criteria

This systematic review included all available original publications about the clinical outcomes and complications of NCCM patients who were receiving ICD treatment. The study was excluded if it did not contain results or complications, or if the publication was a review manuscript or an editorial.

### Search strategy

On 1 November 2021, the databases Embase, MEDLINE, Web of Science, and Cochrane were searched (search terms are provided in the Supplementary Material Text). Inclusion and exclusion criteria were defined a priori. No publications were excluded based on their publication date. First, the publications were screened based on title and abstract. Secondly, Full texts were reviewed in an unblinded standardized manner, performed by two authors in the event of an initial disagreement to include a study. There were duplicates discovered and removed. When two or more publications presented overlapping data, the one with the most patients was chosen. A manual search of the reference lists of the studies reported was also conducted, and the references were appraised using the same inclusion and exclusion criteria.

### Data extraction

Selected studies were checked and relevant patient characteristics, established risk factors for SCD, and length of follow-up were reported. The following baseline characteristics were extracted: gender, type of prevention, left ventricular ejection fraction (LVEF), family history of SCD, ventricular tachycardia (VT), heart failure, and ICD type. The clinical outcomes extracted were cardiac mortality, noncardiac mortality, SCD, heart transplant, appropriate ICD therapy, and inappropriate ICD therapy.

Clinical outcomes concerning the complications included lead malfunction, infection, lead displacement, lead revision, pneumothorax, and any other complications. All reported ICD-related complications were included in the outcome parameter “any complications,” except for inappropriate ICD intervention. No time restriction for complications was used; both early and late complications were included in the analysis.

### Statistical analyses

SPSS version 27 (SPSS Inc, Chicago, IL) was used to document extracted data. Continuous variables were reported as mean and categorical variables were summarized as percentages. Heterogeneity among the studies was assessed using the *Q* test and *I*^2^ index. The random-effects model was used to calculate the summary estimates of the outcome data. From the pooled data, summary estimates of patient characteristics and risk factors for SCD were calculated. Meta-analysis of the outcome data was performed; weighted event rates and weighted annualized event rates were calculated. Forest plots were constructed using the method of Neyeloff et al. [7].

## Results

### Search results

**Fig. 1:**
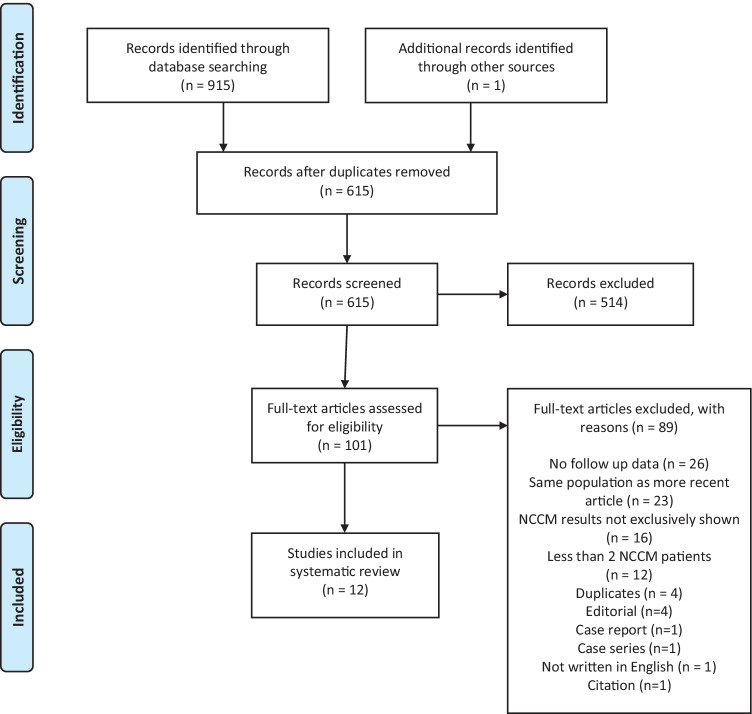
PRISMA flowchart of the literature search and study selection

The systematic literature search resulted in 915 articles, of which 914 were found through database searching and 1 additional article was identified through manual searching the reference lists. After removing duplicates, screening title and abstract, and assessing full-text articles for eligibility, a total of 12 studies met the inclusion criteria (Fig. 1) [8–19]. Twenty-three studies were excluded with the reason being a less recent serial publication. Seven studies were included in the meta-analysis, with Stanton et al. as not estimable because of zero events [8–11, 14, 18, 19]. The 5 remaining studies were excluded because the follow-up duration of NCCM patients in specific was not available or the outcome of (in)appropriate therapies was not available.

In total, 7 studies (58%) reported on a population with NCCM patients with an ICD for primary and/or secondary prevention of SCD [5, 8, 9, 12, 13, 15, 18]. One study reported NCCM patients with secondary arrhythmogenic right ventricular cardiomyopathy (ARVC) [19], one reported NCCM and cardiac implantable electronic devices (CIED) [11], one reported all cardiomyopathies (CMP) and ICD [16], one study reported pediatric CMP and ICD [17], and one reported with genetic heart disease (GHD) and ICD [14].

### Patient characteristics

The baseline characteristics are presented in Table 1. There were 275 patients (mean age 38.6 years; 47% women) with NCCM and ICD implantation. Most of the patients received an ICD for primary prevention of SCD (66%). Risk factors for SCD were left ventricular dysfunction, family history of SCD (29%), ventricular tachycardia (19%), and heart failure (55%). Only 4 studies reported information on ICD type [5, 9, 16, 18]. The majority of the group received a single chamber ICD (46%), followed by dual chamber ICD (30%), biventricular ICD (21%), and subcutaneous ICD (3%).

**Table 1:** Summary of the studies reporting ICD therapy in patients with noncompaction cardiomyopathy

													**ICD type**			
**Author**	**Year**	**Region**	**Population**	***n***	**Mean age (y)**	**Women (%)**	**Primary prevention (%)**	**Secondary prevention (%)**	**LVEF (%)**	**FH of SCD (%)**	**VT (%)**	**Heart failure (%)**	**Single chamber ICD (%)**	**Dual chamber ICD (%)**	**Biventricular ICD (%)**	**Subcutaneous ICD (%)**
Stanton et al. [8]	2009	United States	NCCM & ICD	11	NA	NA	NA	NA	41	NA	27	NA	NA	NA	NA	NA
Kobza et al. [9]	2010	Switzerland	NCCM & ICD	30	48	30	60	40	31	NA	NA	53	46	37	17	0
Caliskan et al. [10]	2011	the Netherlands	NCCM & ICD	44	42	50	73	27	NA	30	11	48	45	25	30	0
Stöllberger et al. [11]	2011	Austria	NCCM & CIED	15	66	20	60	40	NA	NA	NA	73	NA	NA	NA	NA
Engberding et al. [12]	2012	Germany	NCCM & ICD	24	NA	NA	NA	NA	< 35	NA	NA	NA	NA	NA	NA	NA
Favaloro et al. [13]	2012	Argentina	NCCM & ICD	40	NA	30	NA	NA	32	NA	NA	NA	NA	NA	NA	NA
Mcgriff et al. [14]	2013	United States	GHD & ICD	39	50.7	NA	82	18	37.7	NA	NA	NA	NA	NA	NA	NA
Galizio et al. [15]	2015	Argentina	NCCM & ICD	43	NA	NA	NA	NA	NA	NA	0	NA	NA	NA	NA	NA
Ertuğrul et al. [16]	2016	Turkey	CMP & ICD	3	1.1	NA	33	67	NA	NA	NA	NA	0	0	0	100
Migliore et al. [17]	2016	Italy	Pediatric CMP & ICD	4	19	0	75	25	NA	25	25	NA	NA	NA	NA	NA
Sohns et al. [18]	2019	Germany	NCCM & ICD	18	43.5	33	33	67	35	NA	50	61	56	33	11	0
Lutokhina et al. [19]	2020	Russia	ARVC with NCCM & ICD	4	NA	NA	NA	NA	NA	NA	NA	NA	NA	NA	NA	NA
**Summary estimate (12 cohorts)**				275	38.6	47	66	34		29	19	55	46	30	21	3

*ARVC* arrhythmogenic right ventricular cardiomyopathy, *CMP* cardiomyopathy, *FH* family history, *GHD* genetic heart disease. *LVEF* left ventricular ejection fraction. *NA* not available. *VT* ventricular tachycardia

### ICD interventions and outcome

**Fig. 2:**
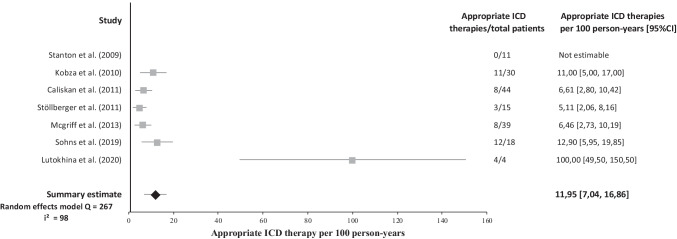
Forest plot of appropriate ICD therapies per 100 person-years in NCCM patients

**Fig. 3:**
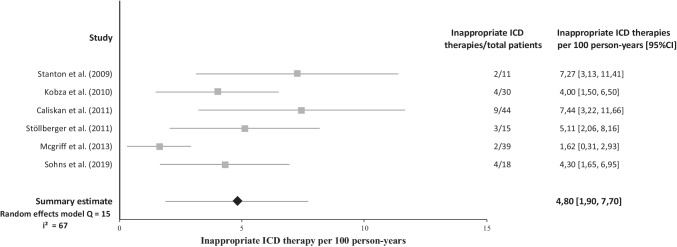
Forest plot of inappropriate ICD therapies per 100 person-years in NCCM patients

During a mean follow-up of 3.1 years, 68 patients (25%) experienced an appropriate ICD therapy (Table 2). Data on inappropriate ICD therapy was described in 9 studies. Thirty-three patients (16%) experienced an inappropriate ICD therapy. The appropriate ICD therapy rate was 11.95 per 100 person-years (Fig. 2), while the inappropriate ICD therapy rate was 4.8 per 100 person-years (Fig. 3). Mortality data was presented in 8 studies. Cardiac mortality was reported in 9 patients (6%). The cardiac mortality rate was 2.37 per 100 person-years and was based on data from 5 studies, with Lutokhina et al. not being estimable because of zero events [5, 9, 11, 18, 19]. Non-cardiac mortality was reported in 4 (4%) patients. Sudden cardiac death was described in 7 studies and no patients were reported with an SCD event. Heart transplantation data was reported in 4 studies and described a heart transplantation procedure in 5 patients (4%).

**Table 2:** Summary of clinical outcome after ICD implantation in noncompaction cardiomyopathy patients

					**Complications**							**Mortality**		
**Author**	**Region**	**Mean follow-up (y)**	**Appropriate therapy (%)**	**Inappropriate therapy (%)**	**Lead malfunction (%)**	**Infection (%)**	**Lead displacement (%)**	**Lead revision (%)**	**Pneumothorax (%)**	**Any (%)**	**Heart transplant (%)**	**Cardiac mortality (%)**	**Non-cardiac mortality (%)**	**Sudden cardiac death (%)**
Stanton et al. [8]	United States	2.5	0	18	NA	NA	NA	NA	NA	NA	NA	NA	NA	NA
Kobza et al. [9]	Switzerland	3.33	37	13	0	0	0	0	0	7	3	3	3	NA
Caliskan et al. [10]	The Netherlands	2.75	18	20	5	2	5	5	5	14	5	5	0	0
Stöllberger et al. [11]	Austria	3.92	20	20	13	7	7	13	0	13	7	13	20	0
Engberding et al. [12]	Germany	NA	21	NA	NA	NA	NA	NA	NA	NA	NA	NA	NA	NA
Favaloro et al. [13]	Argentina	NA	18	23	NA	NA	NA	NA	NA	NA	3	0	0	0
Mcgriff et al. [14]	United States	3.18	21	3	NA	NA	NA	NA	NA	NA	NA	NA	NA	NA
Galizio et al. [15]	Argentina	NA	19	NA	NA	NA	NA	NA	NA	NA	NA	NA	NA	0
Ertuğrul et al. [16]	Turkey	NA	0	0	NA	NA	NA	0	NA	NA	NA	0	0	0
Migliore et al. [17]	Italy	NA	50	0	0	0	0	0	0	0	NA	NA	NA	NA
Sohns et al. [18]	Germany	5.17	67	22	NA	NA	NA	NA	NA	6	NA	22	NA	NA
Lutokhina et al. [19]	Russia	1	100	NA	NA	NA	NA	NA	NA	NA	NA	0	0	0
**Summary estimate (12 cohorts)**		3.1	25	16	4	2	3	4	2	10	4	6	3	0

*NA *not available

### Complications

Six studies (50%), including 114 patients, provided information on the complications of ICD therapy in NCCM patients. Problems with the ICD lead was the most frequent complication type, expressed in 4 studies. Lead malfunctions occurred in 4 (4%) patients, lead revisions in 4 (4%) patients, and lead displacement in 3 (3%) patients. Other complications related to ICD therapy were infection 2 (2%) and pneumothorax 2 (2%). Overall, 10% of the patients experienced any sort of complication during their study follow-up. This includes the previously described complications and less common complications such as T-wave oversensing and psychological complications.

## Discussion

This systematic review and meta-analysis demonstrate that patients with NCCM who are judged to be at increased risk for SCD may significantly benefit from ICD therapy. The pooled analysis, after adjusting for study population and follow-up time, demonstrates an appropriate ICD therapy rate of 11.95 per 100 person-years, and by that, almost certainly preventing SCD. The inappropriate therapy rate was 4.8 per 100 person-years. The cardiac mortality rate was 2.37 per 100 person-years. This study did not find any SCD incidents as a reason of mortality in the study population. Complications were observed in 10% of the patients, with lead-type complications as the most frequent complication; lead malfunctions and lead revisions were present in 4% of patients, lead displacement in 3%, infection in 2%, and pneumothorax in 2%.

Clearly, risk stratification of patients with NCCM is important for management decisions regarding pharmacological therapy and ICD implantation. The 2015 ESC guidelines for the management of patients with ventricular arrhythmias and the prevention of sudden cardiac death recommend the same approach for NCCM as dilated cardiomyopathy (DCM); patients with ventricular arrhythmias require optimum pharmacological treatment, and ventricular arrhythmia triggering causes should be treated. Patients with hemodynamically intolerable ventricular tachycardia or ventricular fibrillations with a life expectancy of more than one year (class 1A recommendation) or symptomatic heart failure with an LVEF of 35 percent or less despite at least three months of optimum pharmacological therapy may consider an ICD.

Ventricular arrhythmias are frequently reported, up to 47%, in patients with NCCM [20]. Kaya et al. suggest prophylactic ICD implantation in patients with non-sustained ventricular tachycardia recordings on Holter in the setting of LV dysfunction (LVEF < 50%), familial history of SCD before 50 years, and early repolarization and/or fragmented QRS on ECG [21].

In clinical practice, an empiric individualized risk stratification for NCCM patients is utilized for decision-making. In secondary prevention of SCD, an ICD is always advised. The studies included in this meta-analysis used the ESC guidelines or comparable decision strategy for the implantation of ICDs in the included patients. In the present analysis, 66% received an ICD for primary prevention of SCD. Secondary prevention ICD implantation was performed in 34%.

Ertuğrul et al. [16] included pediatric patients in their study. They followed the ESC guidelines, which recommend ICD implantation in patients younger than sixteen years after a life-threatening ventricular arrhythmia. ICD implantation as primary prevention in patients younger than sixteen years is recommended when 2 or more major risk factors are present: severe left ventricular hypertrophy, syncope, NSVT, or a family history of sudden death [22].

The present analysis found appropriate ICD therapies were substantially more frequent than inappropriate ICD therapies, with a difference of 7.15 events per 100 person-years. This result, combined with zero events of SCD and a low cardiac mortality rate, shows the positive effects of ICD therapy on preventing SCD in NCCM patients as well as suggesting that no failure to shock occurred in any patient in this study.

However, the inappropriate therapy rate of 4.8 per 100 person-years together with a high incidence of complications is a serious concern, which necessitates a good counseling process before ICD implantation, especially because of the relatively young mean age (38.6 years) of the patient population.

Not all currently known risk factors of SCD were described in each study. However, most patients in this meta-analysis were at increased risk of SCD considering 29% had a family history of SCD, 19% had experienced ventricular tachycardia before ICD implantation, and 4 of the 6 studies, who measured the mean LVEF, had a mean LVEF of 35% or less.

Most of the patients in this pooled analysis received a conventional transvenous single chamber ICD system. Only 1 study included patients who received subcutaneous ICDs (S-ICD). An S-ICD could theoretically bring down the number of complications, considering the most frequent complications in this study were transvenous lead related. Only 3% of the patients in this study received a S-ICD; hence, more information on the use of S-ICD in NCCM is needed.

### Limitations

Firstly, this study included existing abstracts. This resulted in more studies with fewer information and outcomes than full-text articles, such as no description of which guidelines were used to implement an ICD. Secondly, five studies were excluded from the meta-analysis because these studies did not describe a specific follow-up duration for NCCM patients exclusively. Zero event studies could not be included in the meta-analysis. Both the appropriate ICD therapy and cardiac mortality meta-analysis results could therefore be higher in this study than in reality. Next, the decision strategy regarding the ICD implantation strategy was not specified. This meta-analysis did not exclude studies based on age and included one study with pediatric patients. ICD therapy in pediatric patients with NCCM is not well studied and an ICD may have other outcomes in these patients. Finally, the gap between the oldest and latest study is 11 years. Experience with ICD implantation and programming may have improved significantly during that time period.

## Conclusions

Patients with NCCM who are at increased risk of SCD benefit from ICD therapy, with a high appropriate therapy rate and low cardiac mortality rate. Complications were not infrequent and may lead to patient morbidity. Therefore, the advantages and complication types of ICD implantation needs to be explained and discussed with the patient when considering ICD therapy.

## Supplementary information

Below is the link to the electronic supplementary material.

Supplementary file1 (PDF 77 KB)
