# Supplementary material for: Clinical outcomes of implantable cardioverter-defibrillator therapy in noncompaction cardiomyopathy: a systematic review and meta-analysis
Source: Heart Fail Rev. 2022 Jun 10;28(1):241–8. doi: 10.1007/s10741-022-10250-w (PMC9902401; doi:10.1007/s10741-022-10250-w)
Supplement: Supplementary file 1 — Supplementary file1 (PDF 77 KB) [file 10741_2022_10250_MOESM1_ESM.pdf]

Clinical outcomes of implantable cardioverter-defibrillator therapy in noncompaction cardiomyopathy: a systematic review and meta-analysis

Martijn Tukker, BSc[1], Arend F.L. Schinkel[1], MD, PhD, Adem Dereci[1], MD, Kadir Caliskan, MD. PhD, FESC.[1]
[1]*Department of Cardiology, Erasmus MC University Medical Center Rotterdam, Rotterdam, the Netherlands*

Journal name: Heart Failure Reviews

Corresponding author:
Kadir Caliskan, MD, PhD, FESC. Thoraxcenter, Department of Cardiology, Room RG 431. Erasmus MC University Medical Center Rotterdam. Dr. Molewaterplein 40. 3015 GD Rotterdam, The Netherlands.
E-mail: dr.kcaliskan@hotmail.com

Review protocol

Implantable cardioverter-defibrillator (ICD) for prevention of sudden cardiac death (SCD) in patients with noncompaction cardiomyopathy (NCCM): a systematic review and meta-analysis

**Organization, City, Country:**	Erasmus University, Rotterdam, The Netherlands
**Prepared by:**	Martijn Tukker
**Date:**	October 2020
**Senior supervisor:**	Kadir Caliskan
**E-mail corresponding author:**	k.caliskan@hotmail.com
**Sponsor:**	none

Introduction

Non-compaction cardiomyopathy (NCCM) is a cardiomyopathy characterized by hypertrabecularisation of the myocardial wall. The etiology of NCCM is multivariable. A large percentage is familial with a genetic base. NCCM is yet to be classified as a primary cardiomyopathy. The European society of cardiology (ESC) categorized NCCM as an unclassified cardiomyopathy and the American Heart Association describes it as a genetic cardiomyopathy. To diagnose NCCM, echocardiography and MRI can be used.

After diagnosis treatment needs to be considered, because NCCM is associated with a higher risk of congestive heart failure, thrombotic events, supraventricular and ventricular arrhythmias, and sudden cardiac death (SCD). Previous studies reported ventricular arrhythmias in up to 47% and sudden cardiac death in 18% of the adult IVNC patients. Current treatment of patients with NCCM is equal to other cardiomyopathy and consists of oral anticoagulation, managing arrhythmias and heart failure and preventing SCD. To prevent SCD from happening, an implantable cardioverter-defibrillator (ICD) is a rational option. But currently the information on ICD treatment specifically in patients with NCCM is limited. Previous studies often had small population groups and/or short-term outcomes and there is no systematic review written on this subject yet.

Objective

The aim of this systematic review is to investigate the value of implantable cardioverter-defibrillators in patients with NCCM in the prevention sudden cardiac death. Secondly, we will review the complications such as inappropriate shocks, lead malfunction, or displacement.

Methods and Analysis

Eligibility criteria

For the purposes of this literature review, the population, intervention, comparators and outcomes (PICO) framework to inform the review objectives as presented below.

Population	Intervention	Comparison	Outcome
Noncompaction-cardiomyopathy patients	The Implantable cardioverter-defibrillator	No ICD (Medical treatment, no treatment, catheter ablation, other)	sudden cardiac death, (in)appropriate shocks, quality of life, ICD complications etc.

Study characteristics

This systematic review will include the following observational study designs: cohort studies, case control studies and cross-sectional studies. See appendix C for the databases which will be searched. Randomized controlled trials are not likely to be found with this research question. Studies concerning non-compaction cardiomyopathy patients undergoing ICD treatment as the main subject were included.

Searching databases

The table below contains the databases which will be searched with a pre-determined strategy as detailed in Appendix A.

Topic/ field	Database
Cardiovascular health	Embase, medline ALL ovid, Web of science Core Collection, Cochrane CENTRAL register of trials

Types of participants

In this study, we included patients of every age diagnosed with NCCM on the basis of the four morphological criteria mentioned in the introduction.[7] No limits surrounding the participants will be set for gender, ethnicity, number of patients and study setting. We included both children and adults and will analyze them separately. We included patients who are also diagnosed with a different cardiomyopathy besides their NCCM.

Intervention

We included studies which investigated the effects of ICD implantation, in addition to the optimal

medical therapy. For the implantation indication were both Primary and secondary prevention included.

Control group

We included studies with NCCM patients who received medical treatment, no treatment or studies that used patients as their own control group. We also included studies that compared NCCM patients with an ICD implantation to other cardiomyopathy groups with an ICD implantation.

Outcome

A follow-up was needed, with a minimal follow-up length of one day. This is because outcome measures such as sudden cardiac death could happen the day after implementation.

Report characteristics

No limitation was considered for date of publication, date of acceptance of studies and language used. Only published studies or studies that are in press were included.

Exclusion criteria

Case reports, no follow up after ICD implantation, no ICD only therapy (except for CRT and medicine), studies that did not report any of the endpoints of interest.

Assessment of risk of bias and data extraction

Data extraction

To reduce bias, the two reviewers will independently exclude and include studies based on the previously mentioned criteria. Afterwards they compare the decisions and resolve any differences. When a consensus cannot be reached, a third reviewer will be consulted. The data will be collected considering the reasons for exclusion, characteristics of included studies, participants, interventions and outcomes. The following data will be extracted:

Information on Publication: Author, Study name, Journal, Country, date of publication, database

Study Design:

- Enrolment
- Sample Size

- length of follow-up
- method of follow-up
- ICD indication used
- Outcomes measured

Baseline Characteristics of Participants:

- mean age, gender
- NYHA rating of dyspnoea
- LVEF
- Duration of heart failure
- hypertension, diabetes

Intervention-related characteristics

- Number and percentage given ICD
- Number and percentage given CRT
- Number and percentage given ICD and CR

Outcomes

- CV death (incidence, hazard ratio)
- Non-CV death (incidence, hazard ratio)
- Heart transplant
- Appropriate ICD intervention
- Inappropriate ICD intervention
- ICD related complications: lead malfunction, infection, lead displacement, psychological complications, or total complications.

Risk of bias assessment

The risk of bias assessment will be analysed with Preferred Reporting Items for Systematic Reviews and Meta-Analyses (PRISMA) guidelines.

Data synthesis

Only if the data and recourses are suitable, a meta-analysis will be done.

Search strategy

01-11-2021

Database searched	via	Years of coverage	Records	Records after duplicates removed
Embase	Embase.com	1971 - Present	585	561
Medline ALL	Ovid	1946 - Present	165	28
Web of Science Core Collection*	Web of Knowledge	1975 - Present	164	25
Cochrane Central Register of Controlled Trials	Wiley	1992 - Present	1	0
Total			915	614

*Science Citation Index Expanded (1975-present) ; Social Sciences Citation Index (1975-present) ; Arts & Humanities Citation Index (1975-present) ; Conference Proceedings Citation Index- Science (1990-present) ; Conference Proceedings Citation Index- Social Science & Humanities (1990-present) ; Emerging Sources Citation Index (2015-present)

new references: 58

Embase.com

('ventricular noncompaction'/de OR 'left ventricle noncompaction'/de OR 'apical noncompaction cardiomyopathy'/de OR (noncompact* OR non-compact*):ab,ti,kw) AND ('implantable cardioverter defibrillator'/exp OR (((implant* OR internal*) NEAR/6 (defibrillat* OR cardiovert*)) OR icd):ab,ti,kw)

Medline ALL ovid

(Isolated Noncompaction of the Ventricular Myocardium/ OR Noncompaction of Left Ventricular Myocardium with Congenital Heart Defects.nm. OR (noncompact* OR non-compact*).ab,ti,kf.) AND (Defibrillators, Implantable/ OR (((implant* OR internal*) ADJ6 (defibrillat* OR cardiovert*)) OR icd).ab,ti,kf.)

Web of science Core Collection

TS=(((noncompact* OR non-compact*)) AND ((((implant* OR internal*) AND (defibrillat* OR cardiovert*)) OR icd)))

Cochrane CENTRAL register of trials

((noncompact* OR non-compact*):ab,ti,kw) AND ((((implant* OR internal*) NEAR/6 (defibrillat* OR cardiovert*)) OR icd):ab,ti,kw)
